# Malaria prevalence and associated risk factors in Dembiya district, North-western Ethiopia

**DOI:** 10.1186/s12936-021-03906-9

**Published:** 2021-09-17

**Authors:** Mihretu Tarekegn, Habte Tekie, Sisay Dugassa, Yitbarek Wolde-Hawariat

**Affiliations:** 1 Department of Biological Sciences, Faculty of Natural and Computational Sciences, Woldia University, PO. Box, 400, Weldiya, Ethiopia; 2 Department of Zoological Sciences, College of Natural and Computational Sciences, Addis Ababa University, PO. Box, 1176, Addis Ababa, Ethiopia; 3 Aklilu Lemma Institute of Pathobiology, Addis Ababa University, PO. Box, 1176, Addis Ababa, Ethiopia

**Keywords:** Malaria prevalence, Malaria control, *Anopheles*, *Plasmodium*, Malaria risk factors

## Abstract

**Background:**

Ethiopia embarked on combating malaria with an aim to eliminate malaria from low transmission districts by 2030. A continuous monitoring of malaria prevalence in areas under elimination settings is important to evaluate the status of malaria transmission and the effectiveness of the currently existing malaria intervention strategies. The aim of this study was to assess the prevalence of malaria and associated risk factors in selected areas of Dembiya district.

**Methods:**

A cross-sectional parasitological and retrospective survey was conducted in the two localities of Dembiya District, selected based on their long standing history of implementing malaria prevention and elimination strategies. Thin and thick blood smears collected from 735 randomly selected individuals between October and December, 2018 were microscopically examined for malaria parasites. Six years (2012–2017) retrospective malaria data was collected from the medical records of the health centres. Structured questionnaires were prepared to collect information about the socio-economic data of the population. Logistic regression analysis was used to determine a key risk factor explaining the prevalence of malaria. The data were analysed using SPSS version 20 and p ≤ 0.05 were considered statistically significant.

**Results:**

The 6-year retrospective malaria prevalence trend indicates an overall malaria prevalence of 22.4%, out of which *Plasmodium falciparum* was the dominant species. From a total of 735 slides examined for the presence of malaria parasites, 3.5% (n = 26) were positive for malaria parasites, in which *P. falciparum* was more prevalent (n = 17; 2.3%), *Plasmodium vivax* (n = 5; 0.7%), and mixed infections (n = 4; 0.5%). Males were 2.6 times more likely to be infected with malaria than females (AOR = 2.6; 95% CI 1.0, 6.4), and individuals with frequent outdoor activity were 16.4 times more vulnerable than individuals with limited outdoor activities (AOR = 16.4, 95% CI 1.8, 147.9). Furthermore, awareness about malaria transmission was significantly associated with the prevalence of malaria.

**Conclusions:**

Malaria is still a public health problem in Dembiya district irrespective of the past and existing vector control interventions. Therefore, the authorities should work on designing alternative intervention strategies targeting outdoor malaria transmission and improving community awareness about malaria transmission and control methods in the study area. For this, continuous monitoring of vectors’ susceptibility, density, and behaviour is very important in such areas.

## Background

Malaria remains one of a global public health problem affecting an estimated 219 million individuals in 2017, of which more than 92% were reported from a WHO Africa region [1]. From the total global burden of malaria more than 80% were recorded from fifteen countries in sub-Saharan Africa and India [1]. In Ethiopia, malaria accounts for 12% of outpatient consultations and 10% of health facility admissions [2]. More than half of the population in the country (60%) lives in malarious areas, and an estimated 68% of the total population is at risk of malaria infection [2, 3]. The transmission of malaria in Ethiopia is seasonal and unstable, and it varies with altitude and rainfall. In most parts of the country, peak malaria transmission occurs after the main rainy season (July to September). In addition, many areas experience a second minor malaria transmission period following a short rainy season from February to March [2, 3]. Most of the malaria transmissions in Ethiopia occurs in areas below 2000 m.a.s.l, but endemic regions greater than 2000 m are also documented [4, 5].

*Plasmodium falciparum* and *P. vivax* are the dominant malaria parasite species in Ethiopia, which are responsible for 60 and 40% of malaria cases, respectively [6–8]. However, *P. vivax* may be more dominant in different localities of the country with cooler climates [9]. In Ethiopia, *Anopheles arabiensis* is the primary vector of malaria, whereas *Anopheles pharoensis*, *Anopheles funestus* and *Anopheles nili* are secondary vectors in different parts of the country [10].

The government of Ethiopia has made a massive scale-up of malaria control interventions starting from 2005 including diagnostic testing, rapid case treatment using artemisinin-based combination therapy (ACT), prevention and control of malaria among pregnant women using intermittent preventive therapy in pregnancy (IPTp), insecticide-treated bed nets (ITNs), and indoor residual spraying (IRS) [11–14]. For instance, the proportion of individuals living in malarious areas protected by LLINs was increased from nearly zero in 2005 to 51% in 2011, similarly the IRS coverage increased from 10% to 2007 to 38% in 2011 [12]. This has led to a significant reduction of malaria mortality and morbidity in the country [12, 15]. Based on malaria control achievements obtained in the past years the government of Ethiopia has set a goal to eliminate malaria from the country by 2030 [6]. However, the progress towards malaria elimination is hampered because of widespread drug resistance by the parasites, and insecticide resistance in the vectors [15]. This calls for repeated malaria prevalence studies in such areas with high vector control interventions, such as Dembiya District, to design additional malaria control and prevention technologies [6, 16].

Dembia district is one of malaria-endemic area in Ethiopia with long standing implementation of malaria interventions strategies [17]. Over the years, malaria treatment and control measures have resulted in a significant reduction of malaria in the district [18]. However, despite of this considerable progress in malaria control, the disease is still a public health problem in the District [19]. This suggests that a continuous study of the status of malaria prevalence and its determinants in the district are important to design and implement evidence based malaria prevention and control strategies. Therefore, this study aimed to evaluate the retrospective and present trend of malaria transmission, and identifies socio-economic factors for malaria transmission in selected localities of Dembiya District, Northwestern Ethiopia.

## Methods

### Study area description

This study was conducted in Dembiya District found in the North Gondar administrative zone of Amhara regional state. The district is located at 12° 39ʹ N and 37° 09ʹ E. Kola Diba is the capital city of the district, located 750 km north of Addis Ababa and 35 km southwest of Gondar city. The southern part of the district is bordered by Lake Tana. The district has 45 localities (the lowest administrative unit in Ethiopia). The population of Dembiya District was estimated to be approximately 271,000 in 2007, of which 50.9% (138,000) were male and 49.1% (133,000) were females. The majority of the population (91%) lives in rural areas, with most engaging in farming activities; the remaining 9% live in urban areas. The district has 49,528 rural households with 4.3 mean household sizes [20].

The elevation of Dembiya District is between 1500 and 2600 m above sea-level. The agro-ecology of the District is midland (woyna-dega) with respective mean annual minimum and maximum temperature of 11 °C and 32 °C and the mean annual rainfall ranges from 995 to 1175 mm. Information obtained from the district agricultural bureau indicated that the respective proportion of areas considered as plain, mountainous, valleys, and wetland is 87%, 5%, 4.8%, and 3.2%. Out of the total area of the District, 31% is cultivated land, 16% is none cultivable land, 5.6% forest and bush, 12.8% grazing, 8.1% is covered with water, 20.2% swamp and 4.3% is residential areas. The district receives bimodal rainfall, with the short rainy season from March to May and the main rainy season from June to September.

The major crops grown in the District includes teff (*Eragrostis teff*), maize (*Zea mays*), barley (*Hordeum vulgare*), red highland sorghum (*Sorghum bicolor*), and finger millet (*Eleusine Coracana*). Besides, legumes and pulses such as chickpeas (*Cicer arietinum*) and cowpeas (*Vigna unguiculata*) are also grown in the district. They also grow some cash crops like red paper, niger seed (*Guizotia abyssinica*), fenugreek (*Trigonella foenum-graecum*), black cumin (*Nigella sativa*), White cumin (*Cuminum cyminum*), and rice (*Oryza sativa*) with a limited number of farmlands.

**Fig. 1:**
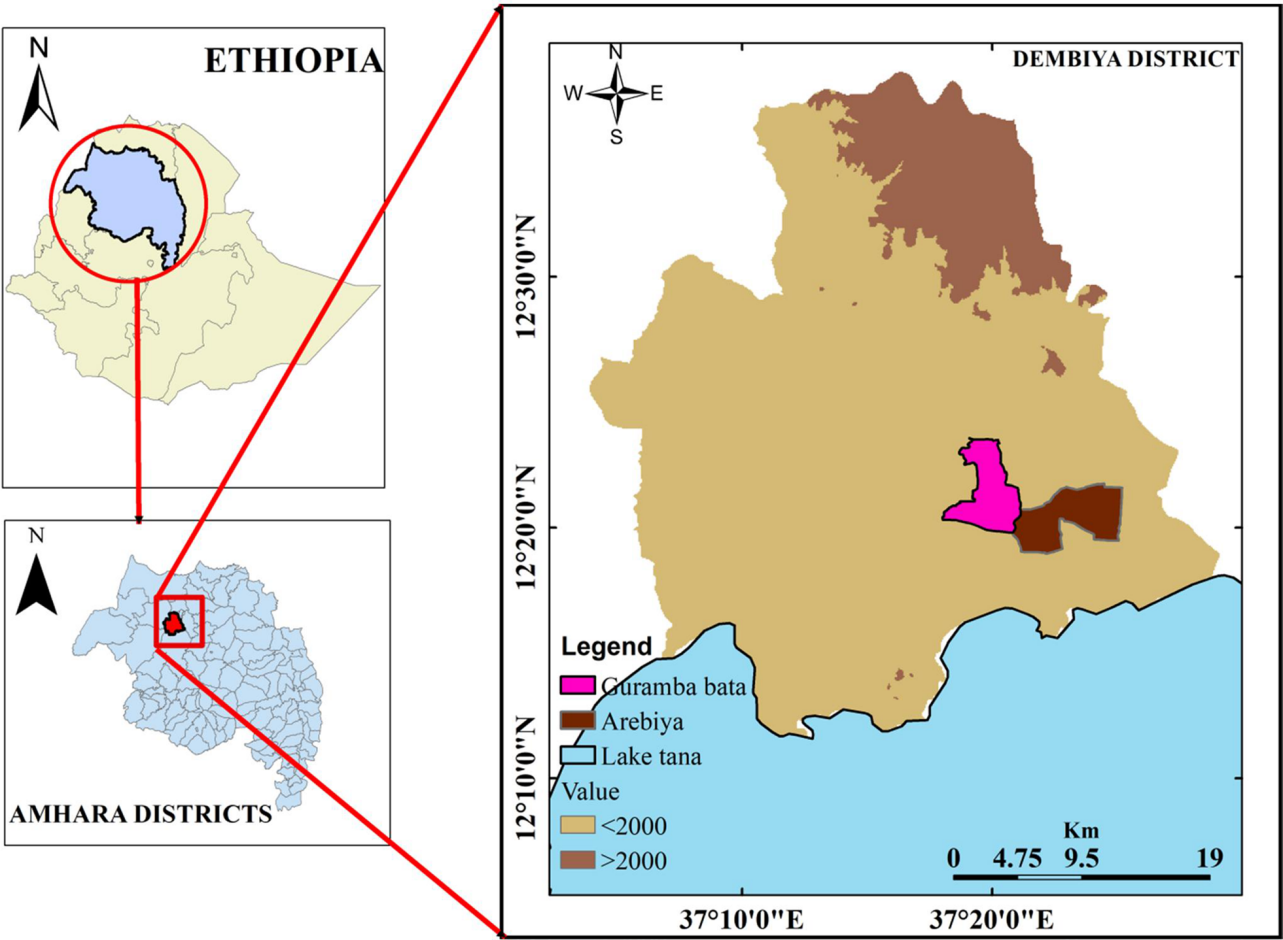
Study area map

Guramba Bata (12° 21ʹ N and 37° 20ʹ E, altitude < 2000 m.a.s.l.) located 7 km away from Kola Diba town. A seasonal river persisted until the end of December serving as one of tributaries to Lake Tana. Guramba Bata has one health post and one health centre, 1113 households with 6008 inhabitants (2974 are male and 3034 are females) in 2017/18 (unpublished health office report) (Fig. 1).

Arebiya (12° 20ʹ N and 37° 22ʹ E, altitude < 2000 m.a.s.l.) is located 17 km away from Kola Diba town. The Megech River is one of the most important rivers serving as a water source during a dry season and drains into Lake Tana. Within 1976 households, the locality has a total of 8632 inhabitants (4298 are male and 4384 are females) in 2017/18. There is one health post in the locality (unpublished health office report) (Fig. 1).

### Study design

A retrospective study was conducted to determine the 6-year (2012 to 2017) of malaria prevalence by reviewing reports at Guramba Bata health centre and Arebiya health post. A cross-sectional parasitological survey was conducted in the two study localities of Dembiya district (Guramba Bata and Arebiya) following the end of the long rainy season (September to October, 2018). The two study sites were selected based on their long history of implementing vector control strategies such as IRS and LLINs (unpublished health office report).

### Retrospective malaria data collection

To assess the retrospective trend of malaria prevalence in the study areas implementing IRS and LLINs vector intervention measures, a 6-year malaria retrospective data (2012–2017) was obtained from Guramba Bata health centre and Arebiya health post. The retrospective malaria prevalence data in the two localities were recorded from microscopic and RDT techniques, which were implemented in the health facilities to confirm the presence of *Plasmodium* parasite in the blood samples.

### Sample size determination for active case detection

The sample size (n) for estimating a population proportion of a small finite population was used to determine the sample size [21].

$$n=\frac{\varvec{n}\varvec{o}}{1+\frac{n0-1}{N}}$$

where n is the minimum sample size for a small population and n_o_ is the sample size for a larger population, N is the population size (N for Guramba Bata = 6008 and N for Arebiya = 8632) and n_o_ is calculated using a single point proportion formula. i.e.

$$no= \frac{{z}^{2 }*p(1-p)}{{d}^{2}}$$

where p is the prevalence of malaria (50%), d is the margin of error (0.05); Z is the standard score corresponds to 1.96.

$$no = \frac{{1.96^{2} 0.5\left( {1 - 0.5} \right)}}{{0.05^{2} }} = 385$$

$${\text{n}}_{2} = \frac{{385}}{{~1 + \frac{{385 - 1}}{{6008}}}} = 365$$

and

$${\text{n}}_{2} = \frac{{385}}{{~1 + \frac{{385 - 1}}{{8632}}}} = 370$$

where n_1_ is the sample size for Guramba Bata study site and n_2_is the sample size for Arebiya study site. 

### Blood sample collection and prevalence study

Blood samples were taken from 365 individuals from Guramba Bata and 370 individuals from Arebiya study sites. These individuals were randomly selected from 160 households, considering 4.3 average persons to household of the Amhara region [20].

Thick and thin smears from finger-prick blood samples were prepared from a total of 735 individuals by well-trained laboratory technicians, from randomly selected households at the end of the rainy season (October–December, 2018). All thin smears were air dried and fixed with methanol in the field. Both thick and thin blood smears were stained with 3% Giemsa solution for 30 min in staining jars in the laboratory. The stained slides were rinsed with tap water and placed in an upright position to dry. The stained thick and thin films were examined with 100x oil immersion objective under a light microscope. The thick blood smear samples were first examined for the presence of *Plasmodium* parasites to determine whether the sample is positive or negative. When samples were positive, thin blood smears were examined for species identification [22].

### Socio-economic survey

A structured questionnaire was prepared to collect information about socio-economic data of the study participants while taking blood samples. Questionnaires were filled by field assistants in consultation with the head of a household during blood sample collection.

### Statistical analysis

The data on retrospective and prospective prevalence of malaria parasites in the two study sites, different age groups, sexes, years and species type were entered using Microsoft excel data sheet and were analysed using SPSS version 20 (Armonk, NY: IBM Corp). Chi squire test was used to compare the difference in frequency of malaria prevalence between independent variables (sex, localities, and age). Association between independent variables with dependent variables was analysed using bivariate logistic regression analysis. Multivariate logistic regression was used to analyse the relative contribution of each independent variable to the dependent variable at p ≤ 0.05.

## Results

### Sociodemographic data

Blood samples for microscopic examination were collected from 735 randomly selected individuals from the two study localities of which 50.3% (n = 370) were from Arebiya and 49.7% (n = 365) were from Guramba Bata. Males comprised 52% (n = 382) while females were 48% (n = 353) of individuals in the sample (Table 1). The age groups, below 15, 5–9, 10–14, and above 15 accounted for 7.3% (n = 54), 18.9% (n = 139), 17.8% (n = 131), and 55.9% (n = 411) of the study participants, respectively. The majority of the study participants were farmers (86.7%; n = 637) and the rest (13.3%; n = 98) were merchants. Most of the study participants (45.9%; n = 341) were not educated. All study participants were from rural areas (Table 1).

**Table 1:** Socio-demographic data of the study participants in the two localities of Dembiya District

Variables	Study sites		Total (%)
	Arebiya	Guramba Bata	
Sex			
Male	200	182	382 (52)
Female	170	183	353 (48)
Total	370 (50.3%)	365 (49.7%)	735
Age			
< 5	30	24	54 (7.3)
5–9	76	63	139 (18.9)
10–14	74	57	131 (17.8)
≥ 15	190	221	411 (55.9)
Total	370	365	735
Occupation			
Farmer	332	305	637 (86.7)
Merchant	38	60	98 (13.3)
Total	370	365	735
Educational status			
No formal education	146	195	341 (46.4)
Primary school attendees	119	104	223 (30.3)
Secondary school attendees	71	48	119 (16.2)
More than secondary	34	18	52 (7.1)
Total	370	365	735 (100)

### Retrospective trends of malaria prevalence

Out of 2157 individuals who visited the two health facilities seeking treatment and suspected to have malaria, 22.4% (n = 484) were positive for malaria parasites (Table 2). Microscopic and RDT results indicated that19.4% (n = 281) individuals in Arebiya and 28.7% (n = 203) individuals in Guramba Bata were infected with malaria parasites during the 6-year period (2012–2017).

**Table 2:** A 6 years retrospective trend of malaria cases in the two localities of Dembiya District (2012–2017)

No. of malaria parasite positive cases (values in parenthesis are %malaria cases)										
Study sites	Arebiya					Guramba Bata				
Age	No. examined	P.f (%)	P.v (%)	Mixed (%)	Total +ve(%)^a^	No. Examined	P.f (%)	P.v (%)	Mixed (%)	Total +ve(%)^b^
< 5	63	2 (3.2)	4 (6.3)	0	6 (9.5)	66	9 (13.6)	8 (12.1)	0	17 (25.8)
6–17	553	23 (4.2)	17 (3.1)	0	40 (7.2)	418	23 (5.5)	8 (1.9)	8 (1.9)	39 (9.3)
18–64	792	187 (23.6)	27 (3.4)	12 (1.5)	226(28.5)	213	114 (53.5)	20 (9.4)	9 (4.2)	143 (67.1)
> 65	41	9 (21.9)	0	0	9 (21.9)	11	0	4 (36.2)	0	4 (36.4)
Total	1449	221 (15.3)	48 (3.3)	12 (0.8)	281 (19.4)	708	146 (20.6)	40 (5.6)	17 (2.5)	203 (28.7)

No. of malaria parasite positive cases (values in parenthesis are %malaria cases)										
Study sites	Arebiya					Guramba Bata				
Sex	No. examined	P.f (%)	P.v (%)	Mixed (%)	Total +ve (%)^c^	No. examined	P.f (%)	P.v (%)	Mixed (%)	Total +ve (%)^d^
Male	931	207 (22.2)	36 (3.9)	11 (1.2)	254 (27.3)	398	110 (27.6)	23 (5.8)	10 (2.5)	143 (35.9)
Female	518	14 (2.7)	12 (2.3)	1 (0.2)	27 (5.2)	310	36 (11.6)	17 (5.5)	7 (2.3)	60 (19.4)
Total	1449	221 (15.3)	48 (3.3)	12 (0.8)	281 (19.4)	708	146 (20.6)	40 (5.6)	17 (2.4)	203 (28.7)

^a^χ^2^ = 111.8, df = 3, p = 0.000; ^b^χ^2^ = 231.7, df = 3, p = 0.000; ^c^χ^2^ = 102.3, df = 1, p = 0.000; ^d^χ^2^ = 21.7, df = 1, p = 0.000

There were significant differences in malaria cases among the age groups in both health facilities (χ^2^ = 111.8, df = 3, p = 0.000; χ^2^ = 231.7, df = 3, p = 0.000). Malaria was more prevalent in individuals between the 18–64 age groups in both health facilities. Malaria parasites were detected in 28.5% (n = 226) individuals in Arebiya health post, and 67.1% (n = 143) individuals in Guramba Bata health centre in the 18–64 age group (Table 2). On the other hand, relatively low number of malaria cases was recorded in the 6–17 years age groups (7.2% in Arebiya health post and 9.3% in Guramba Bata health centre) (Table 2). The difference in malaria cases between sexes were statistically significant in both Arebiya health post (χ^2^ = 102.3, df = 1, p = 0.000) and Guramba Bata study sites health centre (χ^2^ = 21.7, df = 1, p = 0.000). Higher malaria cases were recorded in males (27.3 and 35.9%, respectively) than in females during the 6-year period in both health facilities (Table 2). Furthermore, *P. falciparum* was detected in individuals of all age groups, but it was predominant in individuals between the 18–64 years age group (23.3 and 53.3% in Arebiya and Guramba Bata, respectively). *Plasmodium vivax* was frequently recorded in children less than 5 years of age group in both study localities (Table 2).

**Fig. 2:**
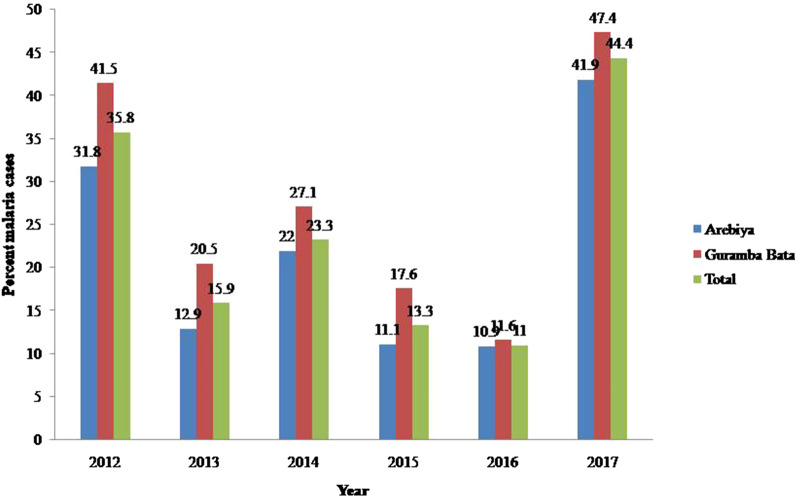
Six-year annual percent malaria cases reported in Arebiya and Guramba Bata (2012–2017)

The lowest malaria cases in Arebiya health post (10.9%; n = 31) were recorded in 2016, while the highest malaria cases (41.9%; n = 72) were encountered in 2017. Similarly, lower malaria cases (11.6%; n = 11) were detected in 2016 with highest (47.4%; n = 72) malaria cases were recorded in 2017 in Guramba Bata health centre (Fig. 2).

**Fig. 3:**
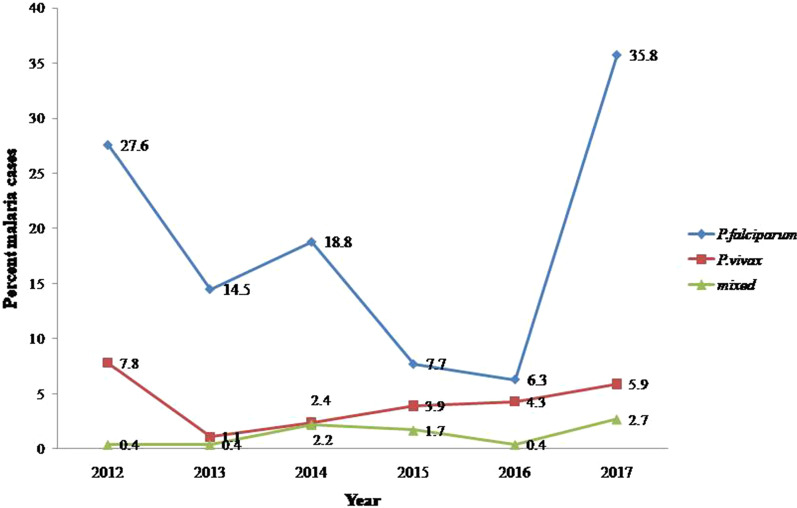
Six-year annual species specific prevalence of malaria parasites reported in Arebiya and Guramba Bata (2012–2017)

*Plasmodium falciparum* was the predominant species in the study sites during the 6 year period (2012–2017) (Fig. 3) with the highest *P. falciparum* malaria cases (35.8%; n = 116) recorded in 2017. *Plasmodium vivax* and mixed infections were recorded in relatively lower magnitude in both sites during the 6-year period (Fig. 3).

### Prevalence of malaria parasites from blood sample examination

Out of the total 735 thick and thin blood smears taken from individuals who participated in the study, 3.5% (n = 26) were positive for malaria parasites. The results from the cross sectional survey indicate that there were no statistically significant difference in percent malaria prevalence between the two localities (χ^2^ = 0.06, df = 1, p = 0.814). The prevalence of malaria infection in Guramba Bata and Arebiya study areas were 3.8% (n = 14) and 3.2% (n = 12) respectively (Table 3). *Plasmodium falciparum* was the predominant malaria parasite (2.3%, n = 17) in the study area, followed by *P. vivax* (0.7%, n = 5), and mixed infections (0.5%, n = 4).

**Table 3:** Prevalence of malaria from the cross-sectional study in the two localities of Dembiya District (October-November, 2018)

Number and proportions of microscopic malaria parasite positive blood samples												
Study sites	Arebiya						Guramba Bata					
Age	No. examined	P.f (%)		P.v (%)	Mix (%)	Total +ve (%)^a^	Age	No. examined	P.f (%)	P.v (%)	Mix (%)	Total +ve(%)^b^
< 5	30	0		0	0	0	< 5	24	1 (4.2)	0	0	1 (4.2)
5–9	76	0		0	0	0	5–9	63	1 (1.6)	0	0	1 (1.6)
10–14	74	1 (1.4)		0	0	1 (1.4)	10–14	57	0	1 (1.8)	0	1 (1.8)
≥ 15	190	6 (3.2)		2 (1.1)	3 (1.6)	11 (5.8)	≥ 15	220	8 (3.6)	2 (0.9)	1 (0.5)	11 (5)
Total	370	7(1.9)		2 (0.5)	3 (0.8)	12 (3.2)	Total	365	10 (2.7)	3 (0.8)	1 (0.3)	14 (3.8)

Number and proportions of microscopic malaria parasite positive blood samples												
Study sites	Arebiya						Guramba Bata					
Sex	No. examined	P.f (%)		P.v (%)	Mix (%)	Total +ve (%)^c^	Age	No. examined	P.f (%)	P.v (%)	Mix (%)	Total +ve (%)^d^
Male	200	6 (3)		1 (0.5)	3 (1.5)	10 (5)	Male	182	4 (2.2)	3 (1.6)	1 (0.5)	8 (4.4)
Female	170	1 (0.6)		1 (0.6)	0	2 (1.2)	Female	183	6 (3.3)	0	0	6 (3.3)
Total	370	7 (1.9)		2 (0.5)	3 (0.8)	12 (3.2)	Total	365	10 (2.7)	3 (0.8)	1 (0.3)	14 (3.8)

^a^χ^2^ = 8.3, df = 3, p = 0.040; ^b^χ^2^ = 2.32, df = 3, p = 0.509; ^c^χ^2^ = 4.3, df = 1, p = 0.039; ^d^χ^2^ = 0.31, df = 1, p = 0.579

The frequency of malaria infection among the age groups was statistically significant in Arebiya study site (χ^2^ = 8.3, df = 3, p = 0.040) (Table 3). Malaria was more prevalent in the age group > 15 years old at this study site (5.8%). Whereas, in Guramba Bata study site the age groups > 15 were more infected with malaria (5%) than the other, but it was not statistically significant (χ^2^ = 2.32, df = 3, p = 0.509) (Table 3). Males were more infected with malaria in Arebiya (5%) and the difference in malaria case between sexes were statistically significant (χ^2^ = 4.3, df = 1, p = 0.039) (Table 3). Similarly, in Guramba Bata study sites males were more infected with malaria (4.4%) than females, though the difference was not statistically significant (χ^2^ = 0.31, df = 1, p = 0.579) (Table 3).

### Malaria risk factor analysis

Bivariate and multivariate analysis indicated that risk factors such as sex, age, outdoor activity in the evening, awareness about malaria transmission, the frequency of LLIN distribution, and application of IRS were significantly associated with malaria prevalence (*P* < 0.05). However, respondent’s occupation, educational level, the last time respondents received IRS were not significantly associated with malaria transmission (*P* > 0.05; Table 4).

**Table 4:** Bivariate and multivariate analysis of factors associated with malaria infection in selected localities of Dembiya District

Variables	Category	Total examined	Positive (%)	OR (95% CI)		
				COR	AOR	p-value
Sex	Female	353	8 (2.3)	1	1	
	Male	382	18 (4.7)	2.13 (0.92–4.97)*	2.58 (1.04–6.41)	0.041
Age	< 5	54	0	1	1	
	5–9	139	1 (0.7)	0.38 (0.02–6.252)*	0.31 (0.02–5.27)	0.417
	10–14	131	3 (2.3)	0.82 (0.07–9.26)	0.53 (0.04–6.48)	0.617
	≥ 15	413	22 (5.3)	2.99 (0.39–22.69)	2.15 (0.27–16.92)	0.466
Occupation	Farmer	637	25 (3.9)	3.96 (0.53–29.58)	4.16 (0.49–35.22)	0.191
	Merchant	98	1 (1)	1	1	
Educational status	No education	341	14 (4.1)	1.07 (0.24–4.85)	1.19 (0.24–5.93)	0.837
	Primary	223	6 (2.7)	0.69 (0.14–3.53)	0.87 (0.16–4.74)	0.873
	Secondary	119	4 (3.4)	0.87 (0.15–4.90)	1.58 (0.25–10.02)	0.629
	> Secondary	52	2 (3.8)	1	1	
Outdoor activity	Yes	571	25 (4.4)	7.46 (1.00–55.50)*	16.42 (1.82–147.85)	0.013
	No	164	1 (0.6)	1	1	
Awareness about transmission	Yes	441	12 (2.7)	1	1	
	No	286	14 (4.9)	1.87 (0.85–4.11)*	3.17 (1.22–8.24)	0.018
Period of receiving last IRS	< 6 month	541	20 (3.7)	1	1	
	6–12 month	194	6 (3.1)	0.83 (0.33–2.10)	1.98 (0.66–5.91)	0.221
Period of receiving last LLIN	< 6month	16	3 (18.8)	1	1	
	6–12 month	15	4 (26.7)	1.58 (0.288–8.61)	3.32 (0.47–23.74)	0.231
	More than year	704	19 (2.7)	0.12 (0.032–0.46)*	0.12 (0.02–0.57)	0.008

* Indicates significant values at p ≤ 0.05

Males were 2.6 times more likely to be infected with malaria than females (AOR = 2.6, 95% CI 1.04, 6.41) and individuals with high outdoor activity were 16.4 times more vulnerable than individuals with limited outdoor activities (AOR = 16.4, 95% CI 1.82, 147.85). Respondents who are not aware of malaria transmission and control were highly infected with malaria than those who were aware of it (AOR = 0.3, 95% CI 0.12–0.82). The last time respondents received LLINs (before a year) was associated with a low level of malaria prevalence in the study area (Table 4).

## Discussion

This study evaluated the 6-year retrospective data of malaria prevalence from health facility records in Guramba Bata and Arebiya localities, where vector control strategies such as IRS and LLINs have been implemented for more than a decade. A snapshot cross-sectional malaria survey was also conducted to determine the level of malaria transmission and the malaria parasites that prevail in the study area. The result of this study showed that malaria is still one of the most important causes of morbidity in the study area. In addition, it was evident that people’s outdoor activities during the night, the community’s low knowledge level about malaria control and prevention and history of receiving LLINs were determining factors which affect malaria transmission in the study area. These imply that malaria elimination programmes need to focus on improving knowledge of the community about malaria prevention and control strategies and look for additional vector control strategies targeting outdoor malaria transmissions in the study area.

The overall percent malaria cases detected in the retrospective malaria study were 22.4% (n = 484) with percent malaria cases peaking towards 2017 (44.4%) despite of the ongoing IRS and LLINs malaria vector control strategies implemented in the study area. This high prevalence of malaria suggests an additional malaria intervention strategies are required to achieve the intended goal of malaria elimination in the study area. A relatively higher percent of malaria cases were reported from a similar retrospective study in the nearby Kola Diba health centre (39.6%) [19], and Serbo health centre (43.8%) [23]. However, this result is higher than a retrospective study conducted in Metema hospital [24] and Kombolcha [25], where the prevalence was 17%, and 7.5%, respectively.

The trend of the 6-year retrospective data indicated that malaria prevalence varied from year to year, with relatively lower malaria cases recorded in 2013, 2015 and 2016. The reduced number of malaria cases during these years could be associated with the accumulated effect of scaled-up malaria intervention strategies in the study area. In contrast to this trend, a relatively higher percentage malaria cases were reported in 2017 (44.4%). The main reason for an increased trend of malaria in 2017 may be the change in *Anopheles* behaviour favouring outdoor biting and resting tendency or due to the development of insecticide resistant vector species or drug resistant *Plasmodium* parasites. In addition it could also be associated with low level of knowledge or perception of the community about malaria prevention and control strategies. This is in agreement with the existing scenarios advocating that malaria remains a public health problem in Ethiopia even though intensive vector intervention strategies were implemented [26].

The overall prevalence of malaria from this cross-sectional study was 3.5% (26/735) and *P. falciparum* was the predominant malaria parasite, this is comparable with the 3.9% prevalence reported from a cross-sectional study conducted in Hawasa town [27]. The result of this prevalence was lower than the 5.3% prevalence of malaria reported in Gondar Town [28], the 5.2% malaria prevalence from Jimma town [29], and 22.1% prevalence among children’s less than 5 years in Arba Minch Zuria [30]. This difference could be attributed to the variation in intensity of vector control strategies, altitude, microclimate, habitat modifications, and community awareness about malaria prevention and control methods.

The species specific prevalence in this study showed that *P. falciparum* was the dominant, whereas *P. vivax* and mixed prevalence holds the second and third position respectively. This is in line with the fact that *P. falciparum* is the dominant parasite in many parts of Ethiopia with altitude below 2000 m above sea level (a.s.l.) [31]. Similar trend of *Plasmodium* parasite distributions were reported from Gilgel-Gibe [32] and children from Northern Ethiopia [33], migrant laborers from North-western Ethiopia [34] and from patients attending Chagni health centre [35]. Retrospective studies from Kola Diba health centre [19], Serbo health centre [23], Metema hospital [24], Kombolcha [25], and Tselemti Woreda [36] and a survey from different part of Ethiopia [37] also support this study finding. However, reports showed that *P. vivax* was the dominant cause of infection in some part of Ethiopia [29, 38, 39]. This variation could be associated with the difference in altitude of the study areas, where *P. falciparum* is dominant in lowland areas (bellow 2000 m) and study period (*P. vivax* is dominant during the dry season), the emergence of drug resistant *P. vivax* to chloroquine, and the relapsing nature of *P. vivax* [40].

The retrospective and prospective studies indicated that malaria infections were more prevalent in males than in females. Similar studies indicated that males were more infected with malaria than females in different part of Ethiopia [41–44] and in Kenya [45]. It was presumed that individual behaviors, environmental and socio-economic factors contribute to transmission of malaria in Ethiopia [46, 47]. Likewise, malaria was more prevalent in individuals above the age group of ≥ 15.This is in agreement with a retrospective study conducted in Kombolcha [25] and Kola Diba health centres [19]. Male individuals at these productive ages are actively involved in outdoor activities such as agriculture and cattle herding, in the evening, which makes them vulnerable to outdoor *Anopheles* mosquito biting. Male individuals usually spend the night outside the house tending cattle in the study sites. These outdoor activities at night were predictors associated with malaria transmission in the study areas. Similarly, different reports indicated that outdoor activities in the evening contributed to high malaria transmission [44, 46] mainly due to the fact that individuals with outdoor activities are exposed to outdoor biting by *Anopheles* mosquitoes [48]. During this study, a substantial number of children were also infected with *Plasmodium* parasites, which indicates the endemicity of malaria in the study area.

The current study showed that poor awareness and knowledge about malaria prevention and control contributed to the prevailing malaria transmission in the study area. Similar reports indicated that awareness about malaria was associated with malaria transmission in Ethiopia [44] and Kenya [45]. This urges for a continuous need to educate and increase awareness of the local communities about malaria transmission towards improved malaria prevention and control strategies.

## Conclusions

This study showed that despite the long standing implementation of vector control strategies such as LLINs and IRS in Dembia district, Northwestern Ethiopia, malaria remains one of the most important health problems in the community. The total prevalence of malaria parasites from a retrospective and prospective study was 22.4 and 3.8% respectively, where *P. falciparum* was found to be the dominant *Plasmodium* species in the study area. Factors such as low knowledge level about malaria prevention and control, outdoor activity during the evening and history of access to LLINs are risk factors for sustaining malaria prevalence in the study area. Therefore, the national malaria elimination program should incorporate additional malaria prevention and control strategies targeting outdoor malaria transmission. Moreover, community health education package about malaria prevention and control should be a part of malaria elimination strategy in the study area. Further studies on the ecology, behaviour, insecticide susceptibility and breeding habitat types of malaria vectors are important for understanding of entomological risk factors.

## Data Availability

The data sets supporting the conclusions of this article are provided in the manuscript.

## Abbreviations

LLINs: Long-lasting insecticidal bed nets
IRS: Indoor residual spray
CI: Confidence interval
χ^2^: Chi square
df: Degree of freedom
RDT: Rapid diagnosis test
IPTp: Intermittent preventive therapy in pregnancy
